# Suspected adverse drug reactions of rivaroxaban reported in the United States food and drug administration adverse event reporting system database: a pharmacovigilance study

**DOI:** 10.3389/fphar.2024.1399172

**Published:** 2024-09-06

**Authors:** Jingying Wu, Jianru Wu, Biyu Tang, Xinru Wang, Fenfang Wei, Yi Zhang, Limin Li, Hongqiao Li, Bei Wang, Wenyu Wu, Xiang Hong

**Affiliations:** ^1^ Key Laboratory of Environmental Medicine Engineering, Ministry of Education, School of Public Health, Southeast University, Nanjing, China; ^2^ Shenzhen Institute of Pharmacovigilance and Risk Management, Shenzhen, China

**Keywords:** suspected adverse event, disproportionality analysis, drug safety, pharmacovigilance, rivaroxaban

## Abstract

**Purpose:**

This study aimed to characterize the safety profiles of rivaroxaban-associated suspected adverse events by mining the Food and Drug Administration Adverse Event Reporting System (FAERS).

**Methods:**

A disproportionality analysis of spontaneously reported suspected adverse drug reactions (ADRs) was conducted. The reports in FAERS from 2014 to 2024 were compiled. Frequentist and Bayesian statistics were both applied to calculate drug-AE combinations in system organ classes and preferred-term levels. Reporting odds ratio (ROR), proportional reporting ratio (PRR), the Medicines and Healthcare products Regulatory Agency (MHRA), Bayesian confidence propagation neural network (BCPNN), and multi-item gamma Poisson shrinker (MGPS) methods were analyzed and used to compare the suspected AEs.

**Results:**

Of 77,384 ADR reports, 66,705 (86.20%) were serious rivaroxaban AE reports. The most common age group was above 65 years. The suspected adverse effects of rivaroxaban emerging for system organ classes (SOCs) primarily included “Gastrointestinal disorders”; “Injury, poisoning, and procedural complications”, “Nervous system disorders” and “Vascular disorders”. Ranked by EBGM, the top signal strength of suspected AE signals of rivaroxaban under ROR algorithm at the preferred-term (PT) level were “Haemorrhagic arteriovenous malformation” (*N* = 571, ROR = 756.520, PRR = 754.029, Information Component (IC) = 7.197, Empirical Bayesian Geometric Mean (EBGM) = 146.725), “Gastrointestinal vascular malformation haemorrhagic” (*N* = 197, ROR = 211.138, PRR = 210.950, IC = 6.614, EBGM = 97.923), and “Diverticulum intestinal haemorrhagic” (*N* = 722, ROR = 169.898, PRR = 169.210, IC = 6.458, EBGM = 97.920). Moreover, uncommon but significantly suspected AE signals, such as “Coagulation factor X level increased”, “Basal ganglia haematoma”, and “Proctitis haemorrhagic” were observed. Notably, “Gastrointestinal haemorrhage” (*N* = 13,436, ROR = 80.477, PRR = 74.460, IC = 5.729, EBGM = 53.042), “Upper gastrointestinal haemorrhage”(*N* = 2,872, ROR = 73.978, PRR = 72.797, IC = 5.706, EBGM = 52.198) and “Internal haemorrhage” (*N* = 2,368, ROR = 91.979, PRR = 80.899, IC = 5.813, EBGM = 56.212) exhibited relatively high occurrence rates and signal strengths. From 2014 to 2024, the IC values of rivaroxaban-associated suspected AEs for “Surgical and medical procedures” and “Cardiac disorders” showed an annual increasing trend in the time-span analysis. Based on the various visulization plots, a key discovery is that “Gastrointestinal hemorrhage” emerged as the most significant suspected AE across five algorithms. The exciting finding was that the MGPS algorithm revealed a higher risk of suspected AEs under the “Investigations” category. However, the results of the analyses of the other algorithms at the SOC level were not akin to this. Moreover, the results of signal mining for the three main types of indication populations with adverse drug reactions (ADRs), including Atrial fibrillation, Cerebrovascular accident prophylaxis, and Deep vein thrombosis were shown that “Gastrointestinal haemorrhage”, “Epistaxis”, “Haematuria”, “Rectal haemorrhage”, and “Upper gastrointestinal haemorrhage” were detected as the most common and significant signals of suspected adverse events.

**Conclusion:**

Rivaroxaban has risks of various suspected adverse reactions while providing therapeutic effects and being used widely. Our pharmacovigilance study may provide valuable hints that practitioners should closely monitor occurrences of “Gastrointestinal disorders”, “Injury, poisoning, and procedural complications” and “Nervous system disorders”, and other events in clinical applications. Consequently, it remains to persist in monitoring rivaroxaban, assessing the associated risks in the future.

## Introduction

Rivaroxaban is a direct inhibitor of Factor Xa (FXa), a drug produced by Bayer Pharma AG based in Germany (Perzborn et al., 2010). The drug has an increased selectivity and comprises a small molecule, which is nearly insoluble in water and has a molecular weight of 436 g/mol. It has high binding for plasma proteins (92%–95%) (albumin as the dominant binding substance) (AG. BP, 2016). In 2008, rivaroxaban was the first new oral direct FXa inhibitor to receive authorization for clinical use. This drug can be used for treating individuals with the prophylaxis of venous thromboembolism (VTE) after elective knee or hip replacement surgical intervention (Mueck et al., 2008). Additionally, it is indicated for the prevention of VTE and pulmonary embolism in cases of ischemic stroke and nonvalvular atrial fibrillation (Maximiliano et al., 2023). This drug is administered orally, making it widely used and accepted compared with subcutaneous injections of anticoagulants such as warfarin.

However, many reports claim adverse effects of rivaroxaban in clinical settings. In 2018, a 79-year-old woman who used rivaroxaban for a stroke developed a rash as an adverse reaction (Sasson et al., 2018). At the same time, Licata et al. summarized the published studies and case reports of rivaroxaban-induced hepatotoxicity (Licata et al., 2018). In 2019, rivaroxaban was reported to induce acute interstitial nephritis in a 70-year-old man (Zafar et al., 2019). In 2020, an 82-year-old patient was hospitalized after being prescribed rivaroxaban for atrial fibrillation. The patient developed a drug-induced hypersensitivity syndrome characterized by low-grade fever, a petechial rash on the legs, and acute renal failure (Marcelino et al., 2020). Simultaneously, a case report showed that a patient developed acute thrombocytopenia [an extremely rare adverse drug reaction (ADR)] after using rivaroxaban (He and Bai, 2020). Studies reported that using rivaroxaban could increase the risk of intraocular hemorrhage (Talany et al., 2017), and rivaroxaban combined with other drugs might increase the risk of bleeding (Gruenebaum et al., 2014). A retrospective cohort study including 581,451 patients aged 65 years and older with atrial fibrillation showed that treatment with rivaroxaban was associated with a significantly increased risk of severe ischemia or bleeding time compared with treatment with apixaban (Ray et al., 2021). Therefore, the adverse effects of rivaroxaban are numerous, necessitating a systematic study to provide a reference for the rational use of this drug.

ADRs are hazardous reactions resulting from the use of medical products, placing a burden on people who use these drugs. At present, the healthcare systems of many developed countries take actions to reduce this impact. These actions include educating clinicians and patients, establishing and managing spontaneous adverse event reporting systems and platforms, providing guidelines for ADR management, and manufacturing safer drugs and antidotes (McDonnell and Jacobs, 2002; Curry et al., 2005; Davies et al., 2007; Plumpton et al., 2016; Khalil and Huang, 2020). In particular, a system of spontaneous reporting of adverse events has emerged as a significant basis for the post-marketing surveillance of medicines. The World Health Organization, the United States Food and Drug Administration, and the European Medicines Agency have successively established databases for monitoring ADRs as an effective method of monitoring the safety of new drugs and regularly supervising the effects of old drugs (Doua and Van Geertruyden, 2014; Guan et al., 2022). In line with this, data mining based on the spontaneous reporting of ADR databases has received extensive attention. Data mining is an application of classical epidemiological and statistical methods to describe and analyse the distribution of suspected drug use and effects (occurrence of adverse reactions) within a certain period, and then explore possible associations between the two. It is significant that mining ADR databases to identify risk signals, intervene the possibe drug risk, guarantee the safety and improve the quality of medical treatment. ADR signal detection is thus the most important technical work in ADR monitoring (Arnaud et al., 2017).

Faced with so many complicated adverse reaction reports of rivaroxaban, it is essential to caary out a data mining study based on of the Food and Drug Administration Adverse Event Reporting System (FAERS) to summarize these yearly reports. And even, it is better to update and visualize the adverse event signals associated with rivaroxaban using some new technique. If possible it is be hoped that this study can provide a basis for the safe clinical use and mangement of the drug.

## Materials and methods

### Data source and data processing

The data for this study were obtained from the FAERS database, which is the largest database globally. It has collected billions of spontaneous adverse event (AE) reports from various regions and districts since 2004. The database is updated quarterly and has been freely available to the public. In this study, all American Standard Code for Information Interchange (ASCII) packet data from 41 quarters spanning from 2014 Q1 to 2024 Q1 were extracted and imported into Statistics Analysis System (SAS) 9.4 software for data cleaning and analysis. According to the recommendations of the US FDA and the data description document, the reports were cleaned, de-duplicated and organized. And ADE reports with rivaroxaban as the primary suspected drug (PS) were obtained by entering “rivaroxaban” in the “DRUGNAME” field and “PROD_AI” field. The original report is characterized by a high degree of data structuring and regularity, and a large amount of available information (Carnovale et al., 2018). In the FAERS, AEs are coded at the preferred-term (PT) level of the Medical Dictionary for Regulatory Activities (MedDRA) classification.

### Detection method of signal mining

Disproportionality analysis is one of the most frequently used methods of safety signal detection and consists of two categories: frequentist statistics and Bayesian statistics. On the one hand, Frequentist statistics includes methods such as reporting odds ratio (ROR) and proportional reporting ratio (PRR). Moreover, the Europe Medicines Agency has proposed a method named the Medicines and Healthcare Products Regulatory Agency (MHRA) method to identify signals. On the other hand, Bayesian statistics includes Bayesian confidence propagation neural network (BCPNN) and multi-item gamma Poisson shrinker (MGPS). The frequentist method has its characteristics: the sensitivity of the frequentist method is high, but it easily produces false-positive signals when the number of reports is small. The specificity of the Bayesian method is good; however, signal detection is relatively delayed. Specially, ROR and PRR could identify abnormally higher than expected proportions of AE reporting, thus highlighting the risks associated with the targeted drug, but PRR is its higher specificity compared to ROR. BCPNN excels in integrating multi-source data and performing cross-validation, which can be capable of capturing potential drug-AE associations. The MGPS algorithm is more comprehensive, quantifying AE signals based on considering the number of reports and background risk, which can detect signals from rare events (Jiang et al., 2024; Zhu et al., 2024). Five methods, such as ROR, PRR, BCPNN, MHRA, and MGPS, were used for signal detection in this study to minimize the result bias caused by using a single algorithm alone. When each algorithm was positive according to its own criteria, it was judged indicating suspicious signals. The 2 × 2 cell table used in the disproportionality analysis method is shown in Table 1. In the table, “a” means the number of reports of a specific AE caused by the target drug; “b” means the total number of all other AEs related to the target drug; “c” means the number of reports of the target AEs caused by all other drugs; and “d” means the total number of all other AEs related to all other drugs.

**TABLE 1:** The 2 × 2 cell table used in the disproportionality analysis method.

Item	Number of target adverse event reports	Number of other adverse event reports	Total
Target drug	a	b	a + b
Other drugs	c	d	c + d
Total	a + c	b + d	n = a + b + c + d

### Risk signal criteria

The formulas of each algorithm and standard of signal detection are shown in Supplementary Table S1, (Guan et al., 2022; Shu et al., 2022). Regarding ROR, a ≥3 and the lower limit of two-sided 95% confidence interval (CI) > 1 were the signal criteria. For PRR, the screening criteria were the same as that for ROR. For MHRA, the criteria for an AE defined as a significant signal were PRR >2, *χ* ^2^ > 4, and a >3. Regarding BCPNN, the screening criteria for a significant signal were the lower limit of the CI (IC-2SD) > 0. According to the signal strength level of BCPNN, if IC-2SD ≤ 0, one drug-AE combination was acknowledged as no signal (−); if 0 < IC-2SD ≤ 1.5, a drug-AE combination was recognized as a weak signal (+); if 1.5 < IC-2SD ≤ 3, one drug-AE combination was defined as a moderate signal (++); if IC-2SD > 3, the drug-AE combination was defined as a strong signal (+++). Regarding MGPS, EBGM05 > 2 was denoted as a significant signal.

### Statistical analysis

Descriptive statistics were used to analyze patient and reporter demographics. Means and standard deviations were reported for continuous variables, whereas frequencies and percentages were used for categorical variables. Data management, processing, and analysis were performed using SAS (version 9.4). Figures were illustrated using GraphPad Prism (version 8.2) or R (version 4.2.2).

## Results

### Descriptive analysis

Information on demographic characteristics of all reports associated with rivaroxaban is shown in Table 2. A total of 77,384 rivaroxaban case reports were included in the study between 2014 and 2024. Among these, the proportion of male patients was more than that of female patients (47.74% vs. 46.24%). Further, 42,613 (55.07%) patients were aged ≥65 years. The median age was 72 years. The year with the highest number of reports was 2015 (19.06%). Further, more than half of all reports were shared by consumers (53.84%). The most reported country was the United States (74.38%), and the most common route of administration was oral (98.69%). Additionally, 86.20% of the reports were classified as serious reports. Hospitalization was the most common outcome of adverse effects for patients (60.24%). The indications of the top 60 most frequently reported rivaroxaban-associated are presented in Table 3. Of these, the most common rivaroxaban-associated indication was “Atrial fibrillation” (*n* = 25,886), followed by “Cerebrovascular accident prophylaxis” (*n* = 13,259), “Deep vein thrombosis” (*n* = 12,346), “Pulmonary embolism” (*n* = 6,248) and “Thrombosis prophylaxis” (*n* = 5,834). As the above three indications accounted for more than 60% of the reports, we conducted separate analyses of adverse reaction reports for each of the three indications. The demographic characteristics of three indications’ reports were also shown in Table 2.

**TABLE 2:** Demographic characteristics of AE reports related to rivaroxaban.

Characteristics	Items	Number of the total Reports (%)	Number of the reports related to atrial fibrillation (%)	Number of the reports related to cerebrovascular accident prophylaxis (%)	Number of the reports related to deep vein thrombosis (%)
Gender	Female (%)	35,781 (46.24)	11,121 (43.43)	5,753 (43.42)	5,846 (47.54)
	Male (%)	36,946 (47.74)	13,163 (51.40)	6,992 (52.77)	5,166 (42.01)
	Not Specified (%)	4,657 (6.02)	1,323 (5.17)	504 (3.80)	1,286 (10.46)
Age group	<18 (%)	121 (0.16)	8 (0.03)	1 (0.01)	28 (0.23)
	≥18, <45 (%)	3,988 (5.15)	189 (0.74)	181 (1.37)	1,383 (11.25)
	≥45, <65 (%)	14,545 (18.80)	2,994 (11.69)	2045 (15.44)	3,420 (27.81)
	≥65	42,613 (55.07)	17,242 (67.33)	9,102 (68.69)	4,290 (34.88)
	NotSpecified (%)	16,117 (20.83)	5,174 (20.21)	1920 (14.49)	3,177 (25.83)
Age (y)	Median (Q1, Q3)	72.00 (61.00,80.00)	76.00 (68.00,82.00)	74.00 (67.00,81.00)	63.00 (50.00,74.00)
Reporting year	2014 (%)	2,842 (3.67)	263 (1.03)	1,103 (8.33)	294 (2.39)
	2015 (%)	14,751 (19.06)	5,827 (22.76)	1,679 (12.67)	2,782 (22.62)
	2016 (%)	13,872 (17.93)	4,775 (18.65)	2,842 (21.45)	2,305 (18.74)
	2017 (%)	10,331 (13.35)	3,445 (13.45)	2,184 (16.48)	1733 (14.09)
	2018 (%)	9,220 (11.91)	3,031 (11.84)	1,090 (8.23)	1,393 (11.33)
	2019 (%)	6,483 (8.38)	2,264 (8.84)	814 (6.14)	963 (7.83)
	2020 (%)	13,378 (17.29)	3,885 (15.17)	3,216 (24.27)	2,188 (17.79)
	2021 (%)	2,237 (2.89)	799 (3.12)	197 (1.49)	264 (2.15)
	2022 (%)	2,189 (2.83)	768 (3.00)	94 (0.71)	227 (1.85)
	2023 (%)	1745 (2.25)	550 (2.15)	30 (0.23)	149 (1.21)
	2024Q1 (%)	336 (0.43)	—	—	—
Reporter	Consumer (%)	41,661 (53.84)	12,057 (47.08)	8,324 (62.83)	6,687 (54.37)
	Physician (%)	20,428 (26.40)	8,604 (33.60)	2,867 (21.64)	2,975 (24.19)
	Pharmacist (%)	9,449 (12.21)	2,986 (11.66)	1,308 (9.87)	1,496 (12.16)
	Other health-professional (%)	4,826 (6.24)	1,687 (6.59)	630 (4.76)	1,001 (8.14)
	Lawyer (%)	510 (0.66)	170 (0.66)	115 (0.87)	79 (0.64)
	Not Specified (%)	510 (0.66)	103 (0.40)	5 (0.04)	60 (0.49)
Country of the reporters	United States of America (%)	57,562 (74.38)	17,468 (68.22)	10,961 (82.73)	10,469 (85.13)
	Germany (%)	2,947 (3.81)	1,359 (5.31)	630 (4.76)	195 (1.59)
	Japan (%)	2,667 (3.45)	1,312 (5.12)	429 (3.24)	176 (1.43)
	France (%)	2,187 (2.83)	879 (3.43)	189 (1.43)	186 (1.51)
	United Kingdom (%)	1817 (2.35)	709 (2.77)	142 (1.07)	252 (2.05)
Route of administration	Oral (%)	76,368 (98.69)	25,331 (98.92)	13,201 (99.64)	12,201 (99.21)
	Other (%)	1,004 (1.3)	276 (1.08)	48 (0.36)	96 (0.78)
	Transplacental (%)	12 (0.02)	—	—	1 (0.01)
Serious Reports	Serious (%)	66,705 (86.20)	22,195 (86.68)	12,408 (93.65)	10,111 (82.22)
	Non-Serious (%)	10,679 (13.80)	3,412 (13.32)	841 (6.35)	2,187 (17.78)
Outcome of AEs	Hospitalization - Initial or Prolonged (%)	46,616 (60.24)	15,103 (58.98)	9,921 (74.88)	7,025 (57.12)
	Other (%)	24,735 (31.96)	8,692 (33.94)	3,346 (25.25)	3,719 (30.24)
	Death (%)	11,482 (14.84)	3,899 (15.23)	2,897 (21.87)	1,514 (12.31)
	Life-Threatening (%)	3,357 (4.34)	1,327 (5.18)	459 (3.46)	382 (3.11)
	Disability (%)	1,466 (1.89)	637 (2.49)	204 (1.54)	121 (0.98)
	Required Intervention to Prevent Permanent Impairment/Damage (%)	216 (0.28)	91 (0.36)	1 (0.01)	21 (0.17)
	Congenital Anomaly (%)	11 (0.01)	1 (0.00)	0 (0.00)	1 (0.01)

**TABLE 3:** Indications of the top 60 most frequently reported rivaroxaban-associated adverse events.

Indication	Number of reports (%)
Atrial fibrillation (%)	25,886 (33.45)
Cerebrovascular accident prophylaxis (%)	13,259 (17.13)
Deep vein thrombosis (%)	12,346 (15.95)
Pulmonary embolism (%)	6,248 (8.07)
Thrombosis prophylaxis (%)	5,834 (7.54)
Thrombosis (%)	3,905 (5.05)
Anticoagulant therapy (%)	1,359 (1.76)
Embolism venous (%)	1,082 (1.40)
Pulmonary thrombosis (%)	383 (0.49)
Knee arthroplasty (%)	361 (0.47)
Coronary artery disease (%)	337 (0.44)
Atrial flutter (%)	333 (0.43)
Cerebrovascular accident (%)	327 (0.42)
Hip arthroplasty (%)	287 (0.37)
Arrhythmia (%)	203 (0.26)
Peripheral arterial occlusive disease (%)	190 (0.25)
Prophylaxis (%)	185 (0.24)
Cardiac disorder (%)	149 (0.19)
Embolism (%)	127 (0.16)
Cardiovascular event prophylaxis (%)	125 (0.16)
Venous thrombosis (%)	125 (0.16)
Transient ischaemic attack (%)	113 (0.15)
Heart rate irregular (%)	98 (0.13)
Antiphospholipid syndrome (%)	89 (0.12)
Coagulopathy (%)	88 (0.11)
Venous thrombosis limb (%)	84 (0.11)
Myocardial infarction (%)	73 (0.09)
Superficial vein thrombosis (%)	73 (0.09)
Factor V Leiden mutation (%)	70 (0.09)
Knee operation (%)	70 (0.09)
Portal vein thrombosis (%)	69 (0.09)
Pulmonary embolism (%)	65 (0.08)
Hypercoagulation (%)	60 (0.08)
Peripheral vascular disorder (%)	56 (0.07)
Stent placement (%)	56 (0.07)
Cardiac ventricular thrombosis (%)	55 (0.07)
Hip surgery (%)	55 (0.07)
Thrombophlebitis (%)	50 (0.06)
Embolic stroke (%)	45 (0.06)
Orthopaedic procedure (%)	45 (0.06)
Cerebral infarction (%)	44 (0.06)
Hypertension (%)	44 (0.06)
Cardiovascular disorder (%)	36 (0.05)
Arrhythmia prophylaxis (%)	34 (0.04)
Cardiac assistance device user (%)	34 (0.04)
Cardiac failure congestive (%)	34 (0.04)
Cardiac fibrillation (%)	34 (0.04)
Surgery (%)	34 (0.04)
Phlebitis (%)	33 (0.04)
Pulmonary hypertension (%)	32 (0.04)
Cardiac failure (%)	31 (0.04)
Heart valve replacement (%)	31 (0.04)
Ischaemic stroke (%)	31 (0.04)
Jugular vein thrombosis (%)	29 (0.04)
Acute coronary syndrome (%)	28 (0.04)
Aortic valve replacement (%)	28 (0.04)
Coronary arterial stent insertion (%)	28 (0.04)
Atrial thrombosis (%)	27 (0.03)
Blood disorder (%)	27 (0.03)
Arterial thrombosis (%)	26 (0.03)

### The proportion of suspected adverse events under SOC level with rivaroxaban

**FIGURE 1:**
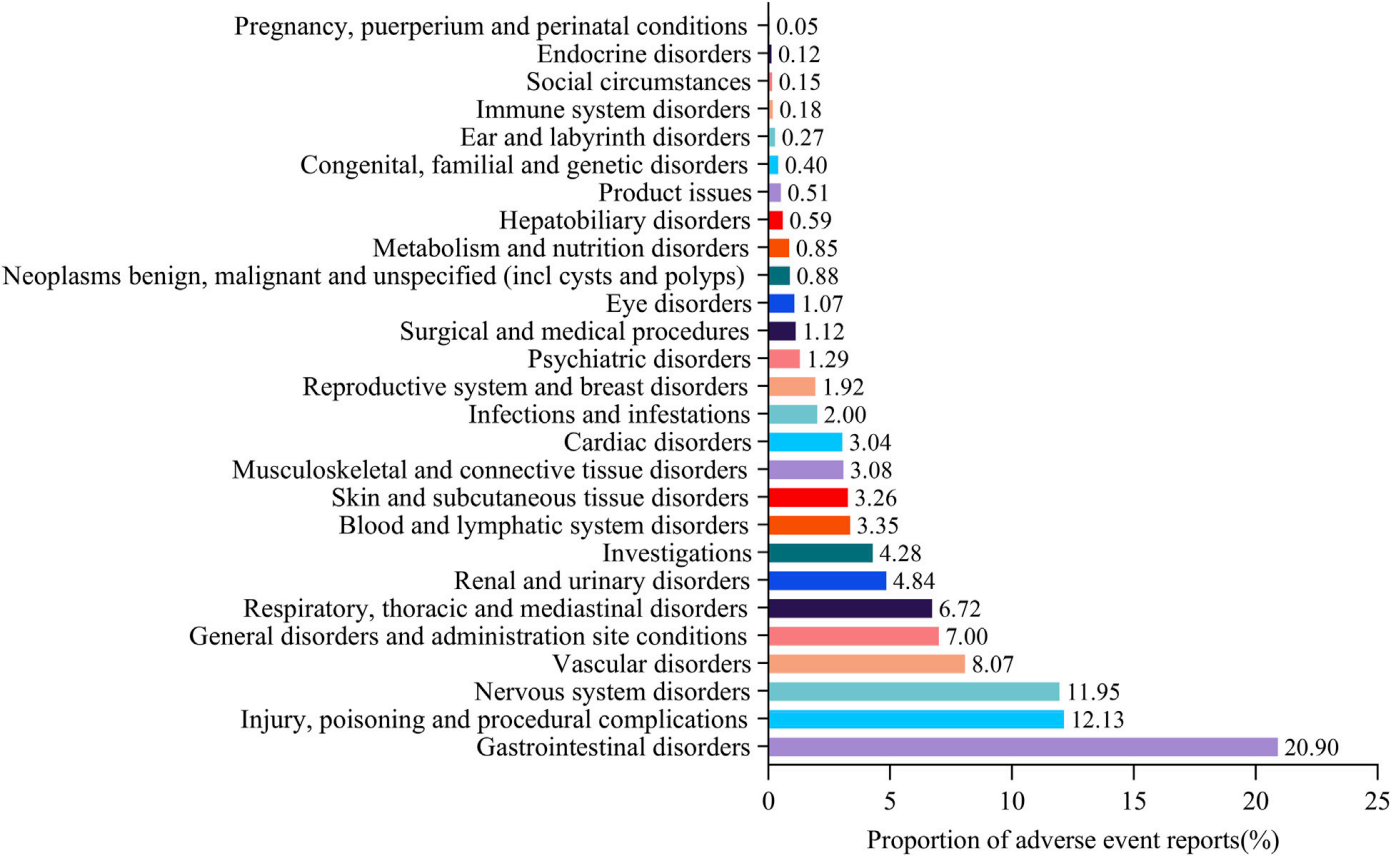
Proportion of suspected adverse event reports under system organ class (SOC) for rivaroxaban.* Proportion of suspected adverse event reports under organ system classification (SOC) = the number of suspected adverse event reports under organ system classification (SOC)/total number of suspected adverse event reports of target drugs.

The proportion of suspected adverse events reports under SOC level was drawn (Figure 1), which was equal to the number of suspected adverse event reports under organ system classification (SOC)/total number of suspected adverse event reports of target drugs. “Gastrointestinal disorders” were associated with the highest number of suspected adverse event reports (20.90%), followed by “Injury, poisoning, and procedural complications” (12.13%), “Nervous system disorders” (11.95%), “Vascular disorders” (8.07%), and “General disorders and administration site conditions” (7.00%). The detailed information was provided in Supplementary Table S2.

### Time trend of different SOCs for rivaroxaban-associated suspected AEs based on their IC and their 95% CIs

This study generated time series analyses of safety signals of different SOCs for investigating the changes in each signal over time. When a graph showed an upward trend and the CI gradually narrowed, it indicated that the signal was stable and strongly correlated with the use of rivaroxaban. The horizontal axis represented the year of the report, whereas the vertical axis represented the information component (IC) value and its 95% CI.

**FIGURE 2:**
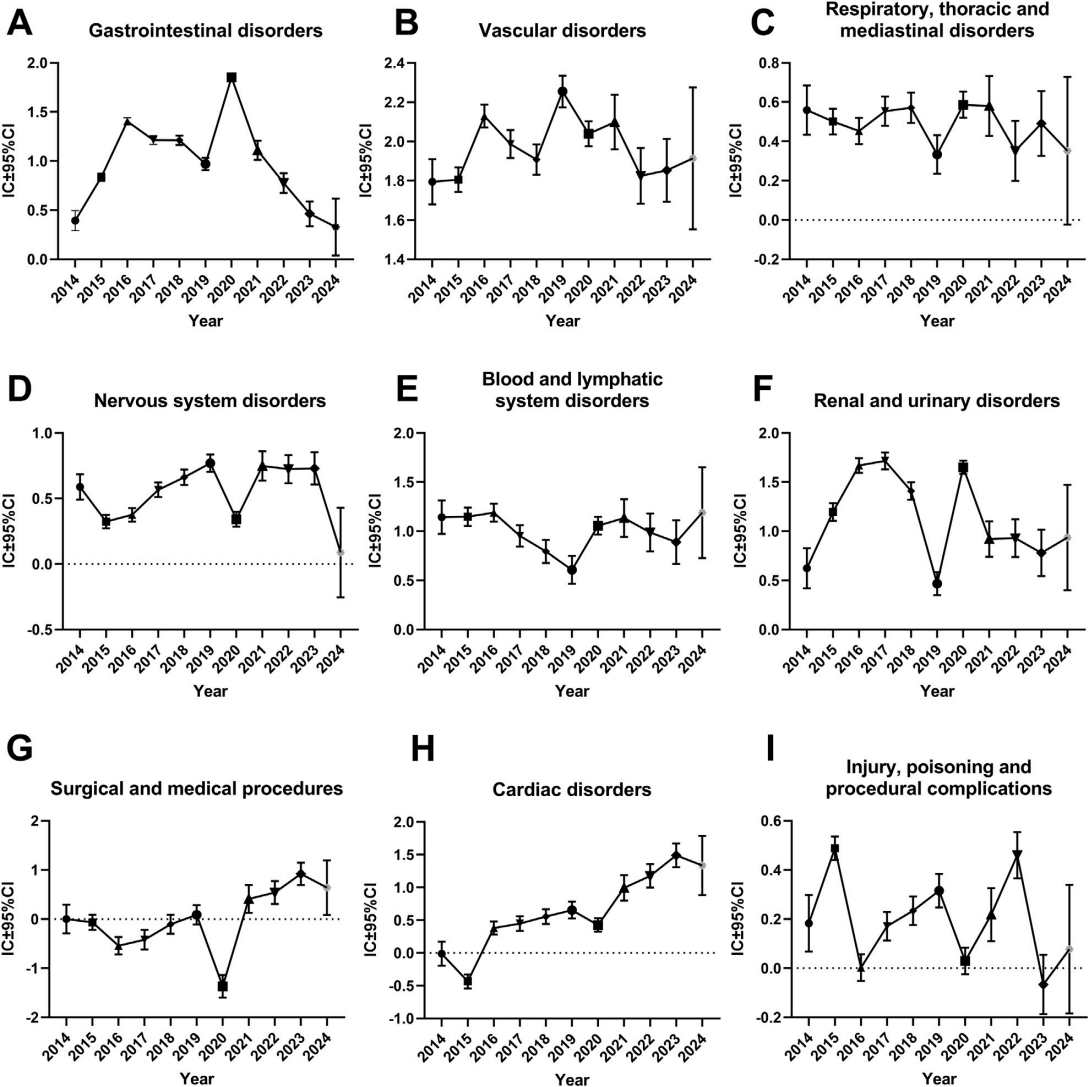
Information component and its 95% confidence interval over time for different system organ classes of rivaroxaban-associated adverse events. **(A)** Gastrointestinal disorders **(B)** Vascular disorders **(C)** Respiratory, thoracic and mediastinal disorders **(D)** Nervous system disorders **(E)** Blood and lymphatic system disorders **(F)** Renal and urinary disorders **(G)** Surgical and medical procedures **(H)** cardiac disorders **(I)** Injury, poisoning and procedural complications. Abbreviations: CI, confidence interval; IC, information component.

As shown in Figures 2G, H, the IC values of rivaroxaban-induced suspected AEs for “Surgical and medical procedures” and “Cardiac disorders” had an augmented trend with the increase in the number of years. Because the 2024 data for this study only included the first quarter, the picture shows a wide range of ICs and their 95% confidence intervals. So, the IC values across “Surgical and medical procedures” and “Cardiac disorders” accumulated gradually, and the range of CI continued to narrow or hold the line from 2014 to 2023. However, the other SOCs did not display this upward trend. Specially, the IC values of other SOCs, such as “Gastrointestinal disorders”, “Vascular disorders”, “Respiratory, thoracic, and mediastinal disorders”, “Nervous system disorders”, “Blood and lymphatic system disorders”, “Renal and urinary disorders” and “Injury, poisoning and procedural complications” were all above 0, suggesting that these SOCs had some suspect signals (Figures 2A–F, I). However, no such obvious hints were found in additional SOCs presented in Supplementary Figure S1 and Supplementary Figure S2. Their IC values and its 95%CI were all under 0, which means as far as BCPNN algorithm was concerned, these SOCs might not have suspect signals from statistical meaning.

### Suspected AE signals associated with rivaroxaban

**FIGURE 3:**
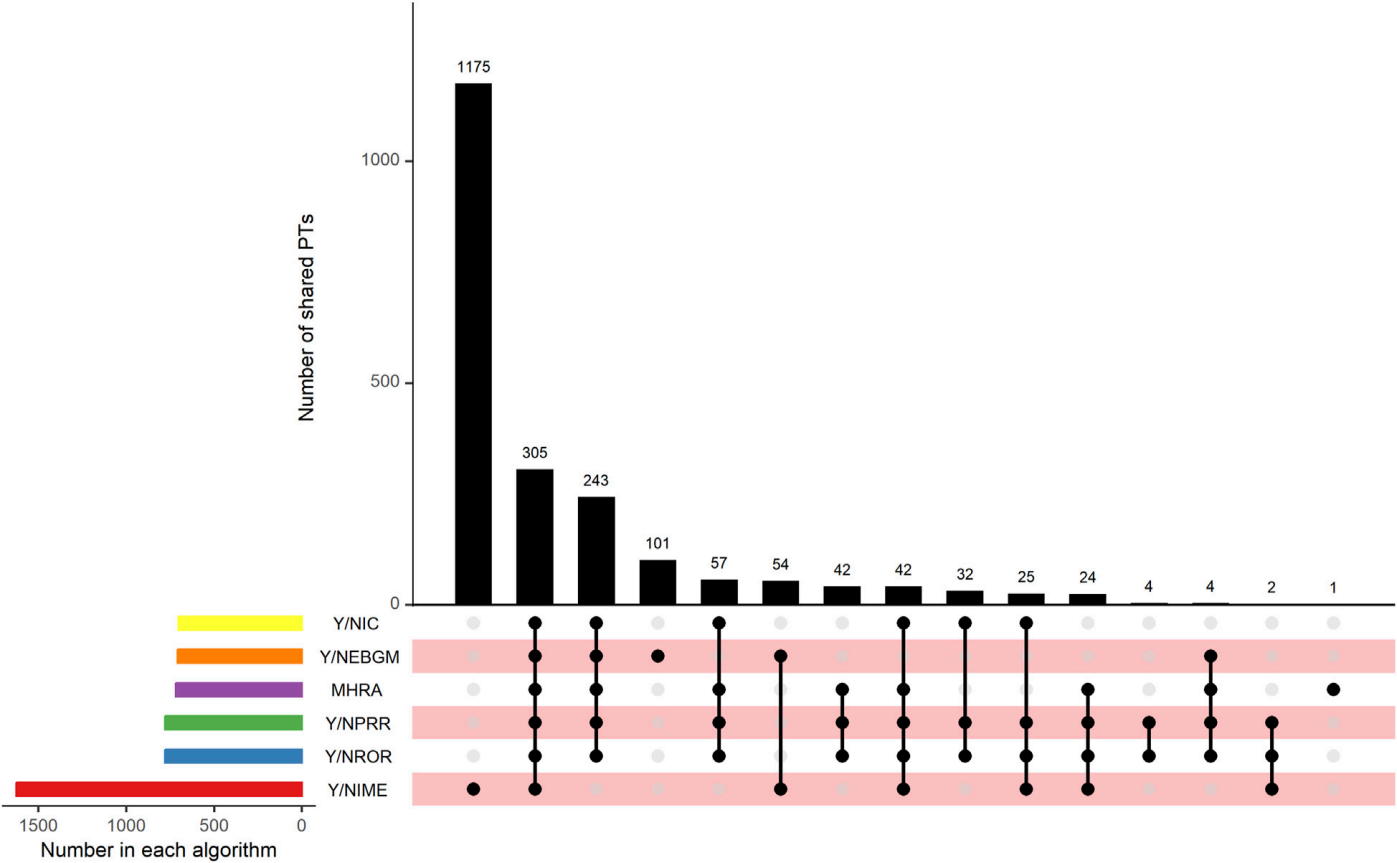
UpSet plot shows the number of PTs in each algorithm. Abbreviations: IC, a value named information component in BCPNN algorithm; BCPNN, Bayesian confidence propagation neural network; EBGM, a value named empirical Bayesian geometric mean in MGPS algorithm; MGPS, multi-item gamma Poisson shrinker; MHRA, a ≥3 a, PRR ≥2, and *χ* ^2^ ≥ 4; PRR, proportional reporting ratio; ROR, reporting odds ratio; IME, important medical event.

This study used five algorithms, including ROR, PRR, MHRA, MGPS, and BCPNN, to detect the suspected AE signals for rivaroxaban at the PT level. There were 177,465 combinations of rivaroxaban-associated AEs reported in the FAERS databas. One report (patient) may report several adverse events, so the data was generally be greater than the number of reports. The ROR and PRR algorithms recognized 780 signals of rivaroxaban-associated AEs. A total of 718 signals of rivaroxaban-associated AEs were identified by the MHRA algorithm. EBGM picked out 707 signals, whereas BCPNN distinguished 704 signals. The top 50 signal strength of suspected adverse events with rivaroxaban under the ROR algorithm are sorted by EBGM at the PT level in FAERS are exhibited in Table 4. The European Medicines Agency has drawn up a list of criteria to differentiate the important medical events (IMEs). Based on the latest version of MedDRA 26.1, IMEs integrated 1,627 PTs. The UpSet diagram provided the number of common and unique elements between five or more groups. Accordingly, we added the IME classification to the five algorithms to draw an UpSet diagram to show the detected signals of suspected adverse events, as shown in Figure 3. Furthermore, 305 PTs were confirmed for important medical events and 278PTs represent non-important medical events by the five methods detected. However, 1,175 were IMEs but they were not detected as suspect signals by five algorithms. Regardless of whether it was a significant medical event or not, it represented the signal that all five algorithms collectively detected, which was 305 + 243 = 548; this number was consistent with the result of the Venn diagrams (Supplementary Figure S3).

**TABLE 4:** The top 50 signal strength of adverse events with rivaroxaban under the ROR algorithm are sorted by EBGM at the PT level in FAERS.

PT	Drug-ADR count	ROR	ROR 95%CI	PRR	PRR 95%CI	Chi-square	IC	IC 95%CI	IC signal strength	EBGM	EBGM 95%CI	IME (important medical event)
Haemorrhagic arteriovenous malformation	571	756.520	627.791–911.646	754.089	625.847–908.61	83,100.380	7.197	6.706–7.03	+++	146.725	121.758–176.811	Y
Coagulation factor X level increased	8	289.499	94.705–884.951	289.486	94.703–884.894	884.582	6.807	1.83–4.296	++	111.956	36.625–342.232	N
Gastrointestinal vascular malformation haemorrhagic	197	211.138	171.907–259.322	210.905	171.736–259.008	19,003.570	6.614	5.782–6.291	+++	97.923	79.729–120.27	Y
Anastomotic ulcer haemorrhage	47	177.206	118.520–264.951	177.159	118.495–264.867	4,159.717	6.492	4.464–5.484	+++	90.007	60.199–134.575	Y
Diverticulum intestinal haemorrhagic	722	169.898	153.487–188.063	169.210	152.899–187.262	62,388.220	6.458	6.163–6.424	+++	87.920	79.428–97.32	Y
Chronic gastrointestinal bleeding	69	145.220	105.788–199.349	145.164	105.755–199.258	5,481.147	6.340	4.821–5.651	+++	80.988	58.997–111.175	Y
CHA2DS2-VASc annual stroke risk high	3	135.699	30.370–606.327	135.696	30.37–606.308	229.207	6.285	0.171–3.705	+	77.969	17.45–348.382	N
Subgaleal haematoma	47	132.904	91.203–193.673	132.869	91.184–193.611	3,546.659	6.267	4.395–5.391	+++	77.033	52.862–112.255	Y
Haemorrhagic erosive gastritis	75	94.932	71.786–125.542	94.893	71.761–125.48	4,570.610	5.968	4.724–5.492	+++	62.590	47.329–82.772	Y
Lower gastrointestinal haemorrhage	1,545	91.220	85.801–96.982	90.435	85.093–96.113	91,122.360	5.922	5.782–5.952	+++	60.630	57.028–64.459	Y
Basal ganglia haemorrhage	176	89.788	74.941–107.576	89.700	74.876–107.458	10,320.660	5.914	5.245–5.746	+++	60.300	50.329–72.247	Y
Dieulafoy’s vascular malformation	62	88.358	65.216–119.712	88.327	65.198–119.662	3,597.008	5.899	4.526–5.365	+++	59.680	44.049–80.858	N
Cerebral ventricle collapse	21	82.607	49.299–138.422	82.598	49.295–138.4	1,162.229	5.833	3.294–4.709	+++	57.022	34.03–95.55	Y
Cerebral ventricular rupture	44	81.253	56.932–115.964	81.233	56.922–115.929	2,406.396	5.817	4.162–5.148	+++	56.372	39.499–80.454	Y
Internal haemorrhage	2,368	81.979	78.084–86.069	80.899	77.090–84.896	129,156.000	5.813	5.711–5.848	+++	56.212	53.541–59.016	Y
Mesenteric haemorrhage	17	76.902	43.600–135.641	76.895	43.597–135.623	893.637	5.762	2.992–4.549	++	54.259	30.762–95.704	Y
Thalamus haemorrhage	172	76.534	64.035–91.474	76.461	63.981–91.375	9,004.476	5.756	5.118–5.618	+++	54.044	45.218–64.594	Y
Gastrointestinal haemorrhage	13,436	80.477	78.831–82.157	74.460	73.019–75.929	690,832.900	5.729	5.694–5.753	+++	53.042	51.957–54.15	Y
Post procedural haematuria	29	72.886	47.361–112.167	72.874	47.356–112.144	1,465.485	5.707	3.665–4.864	+++	52.237	33.943–80.39	N
Upper gastrointestinal haemorrhage	2,872	73.978	70.825–77.272	72.797	69.729–76.000	145,064.900	5.706	5.619–5.742	+++	52.198	49.973–54.522	Y
Oesophagitis haemorrhagic	45	72.713	51.447–102.769	72.695	51.437–102.736	2,269.881	5.704	4.138–5.105	+++	52.145	36.895–73.699	Y
Embolic cerebral infarction	113	72.545	58.320–90.241	72.500	58.289–90.175	5,688.637	5.702	4.859–5.472	+++	52.045	41.84–64.74	Y
Haemorrhagic stroke	1,207	72.464	67.777–77.475	71.978	67.345–76.93	60,444.720	5.694	5.54–5.729	+++	51.777	48.428–55.358	Y
Gastritis haemorrhagic	212	69.569	59.376–81.512	69.487	59.314–81.405	10,339.630	5.658	5.131–5.578	+++	50.483	43.086–59.15	Y
Gastrointestinal polyp haemorrhage	113	69.114	55.644–85.845	69.071	55.615–85.783	5,486.292	5.651	4.825–5.436	+++	50.264	40.468–62.432	Y
Adrenal haemorrhage	82	68.717	53.289–88.613	68.686	53.269–88.565	3,964.458	5.646	4.615–5.331	+++	50.061	38.821–64.555	Y
Renal haemorrhage	308	63.290	55.585–72.062	63.181	55.499–71.927	13,970.760	5.557	5.171–5.54	+++	47.087	41.355–53.614	Y
Duodenitis haemorrhagic	21	60.317	36.808–98.840	60.310	36.805–98.824	918.639	5.507	3.216–4.597	+++	45.482	27.755–74.531	Y
Splenic haematoma	42	58.921	41.594–83.465	58.907	41.587–83.44	1803.600	5.482	3.974–4.958	+++	44.684	31.544–63.298	Y
Peptic ulcer haemorrhage	76	58.788	45.382–76.154	58.763	45.367–76.115	3,257.380	5.479	4.461–5.196	+++	44.602	34.431–57.777	Y
Atrial thrombosis	274	55.044	48.079–63.018	54.961	48.014–62.912	11,134.410	5.406	5.009–5.396	+++	42.388	37.025–48.528	Y
Chest wall haematoma	45	54.292	38.908–75.758	54.279	38.901–75.734	1810.291	5.392	3.996–4.942	+++	41.984	30.087–58.583	Y
Basal ganglia haematoma	6	54.280	21.798–135.166	54.279	21.798–135.159	241.371	5.392	1.384–3.832	+	41.984	16.86–104.545	Y
Pericardial haemorrhage	277	53.626	46.894–61.325	53.544	46.83–61.221	11,021.660	5.377	4.987–5.371	+++	41.545	36.33–47.509	Y
Post procedural haematoma	198	52.895	45.147–61.974	52.838	45.104–61.898	7,793.791	5.362	4.869–5.322	+++	41.121	35.097–48.179	N
Oesophageal ulcer haemorrhage	50	52.005	37.971–71.228	51.991	37.963–71.203	1942.374	5.344	4.063–4.959	+++	40.609	29.65–55.619	Y
Epidural anaesthesia	3	49.345	13.766–176.88	49.344	13.766–176.874	111.645	5.285	0.262–3.509	+	38.985	10.876–139.743	N
Cerebellar haemorrhage	180	49.096	41.637–57.890	49.047	41.602–57.825	6,665.437	5.278	4.767–5.239	+++	38.800	32.906–45.75	Y
Haemorrhoidal haemorrhage	592	48.275	44.087–52.862	48.118	43.953–52.676	21,577.730	5.256	5.037–5.298	+++	38.219	34.903–41.85	N
Haemorrhagic hepatic cyst	12	47.202	25.005–89.102	47.199	25.004–89.093	430.370	5.234	2.411–4.179	++	37.640	19.94–71.053	Y
Proctitis haemorrhagic	9	46.527	22.365–96.793	46.524	22.364–96.785	318.909	5.218	1.994–4.012	++	37.213	17.887–77.416	Y
Incision site haematoma	22	46.290	28.98–73.938	46.284	28.978–73.925	776.172	5.212	3.184–4.508	+++	37.060	23.202–59.195	N
Diverticulitis intestinal haemorrhagic	39	45.829	32.251–65.125	45.820	32.246–65.107	1,364.316	5.200	3.774–4.776	+++	36.763	25.87–52.241	Y
Retroperitoneal haemorrhage	233	45.535	39.439–52.572	45.476	39.394–52.497	8,099.600	5.192	4.78–5.193	+++	36.543	31.651–42.19	Y
Exsanguination	11	45.235	23.363–87.584	45.232	23.362–87.576	380.638	5.185	2.28–4.116	++	36.386	18.792–70.45	Y
Gastric ulcer haemorrhage	556	44.927	40.939–49.303	44.789	40.823–49.141	19,081.550	5.174	4.951–5.219	+++	36.100	32.895–39.617	Y
Duodenal ulcer haemorrhage	280	44.159	38.748–50.325	44.091	38.695–50.239	9,481.634	5.156	4.799–5.175	+++	35.647	31.28–40.625	Y
Urogenital haemorrhage	29	43.731	29.151–65.605	43.724	29.148–65.591	975.041	5.146	3.462–4.617	+++	35.409	23.603–53.12	Y
Embolic stroke	372	43.067	38.460–48.227	42.979	38.389–48.119	12,325.850	5.126	4.836–5.163	+++	34.921	31.185–39.105	Y
Haemorrhagic cerebral infarction	66	42.816	32.740–55.993	42.800	32.73–55.968	2,178.942	5.121	4.144–4.914	+++	34.804	26.613–45.515	Y

**FIGURE 4:**
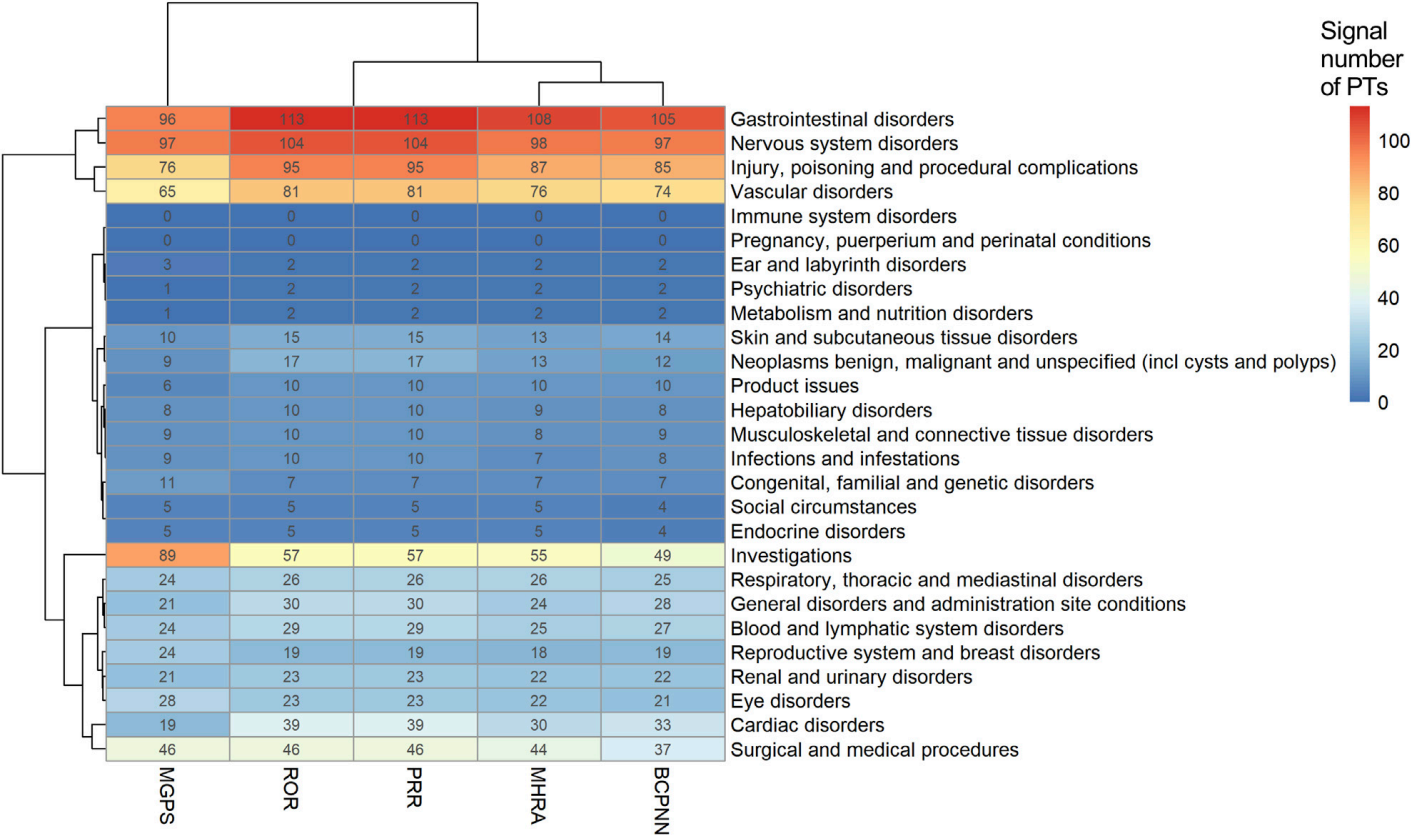
Signals of adverse events detected using ROR, BCPNN, MHRA, and MGPS algorithms at the SOC level. Abbreviations: MGPS, multi-item gamma Poisson shrinker; ROR, reporting odds ratio; PRR, proportional reporting ratio; MHRA, a ≥3, PRR ≥2, and *χ* ^2^ ≥ 4; BCPNN, Bayesian confidence propagation neural network.

The heat map plot was drawn to reflect the signals of suspected AEs detected by ROR, BCPNN, MHRA, and MGPS algorithms at the SOC level. The horizontal axis denotes different algorithms, the vertical axis denotes different SOC classifications, and the color shades denote the signal numbers of PTs. A darker color indicates more signal numbers of PTs. It was obvious that “Gastrointestinal disorders” had the maximum signal numbers of PTs regardless of the algorithms (Figure 4). All five methods found that the risk of AEs was the highest for “Gastrointestinal disorders”, followed by “Nervous system disorders” and “Injury, poisoning and procedural complications”. However, the risk of ADR was low for “Pregnancy, puerperium and perinatal conditions”, “Immune system disorders”, and “Psychiatric disorders”. Interestingly, the MGPS algorithm found a higher risk of AEs under “Investigations” category, but the results of the analyses of the other methods were not consistent with this. The “Investigations” included “Heamatology investigations (including blood groups)”, “Cardiac and vascular investigations (excluding enzyme tests)”, “Renal and urinary tract investigations and urinalyses”, and so on. The Venn diagrams of rivaroxaban were designed to find similar numbers of suspected AE signals. A total of 548 signals were collectively identified using five algorithms, as shown in Supplementary Figure S3. Of these, the ROR, PRR, and IC methods detected the highest overlap of signals and were therefore considered to be more conservative and reliable. The EBGM method additionally detected the maximum number of signals (*n* = 155) and might be more sensitive. Supplementary Figure S4 shows the percentage of every high-level group term under the “Investigations” category. A volcano plot was developed to investigate the relationship between the ROR, PRR, IC, and EBGM and significant differences to identify rivaroxaban associated with AEs (Supplementary Figure S5). The *x*-axis denotes the logarithm of the reporting ROR or PRR or IC lower limit or EBGM lower limit, and the *y*-axis denotes the negative logarithm of the *P* value calculated using the chi-square test (-ln(*P* value)). Positive values in the direction of the *y*-axis represent significant differences. The colors of the points represent the difference in the logarithm of the number of each ADR. In this scatterplot, the point in the upper right corner indicates a stronger signal. The blue-to-red colors represent the number of times of reported adverse effects. Each label represents a specific PT. Based on the plots, “Gastrointestinal Hemorrhage”, “Upper gastrointestinal haemorrhage”, “Rectal haemorrhage”, “Internal haemorrhage” and “haematuria” were identified as the most significant signals across four algorithms.

### Signals of suspected adverse events associated with atrial fibrillation, cerebrovascular accident prophylaxis and deep vein thrombosis

**FIGURE 5:**
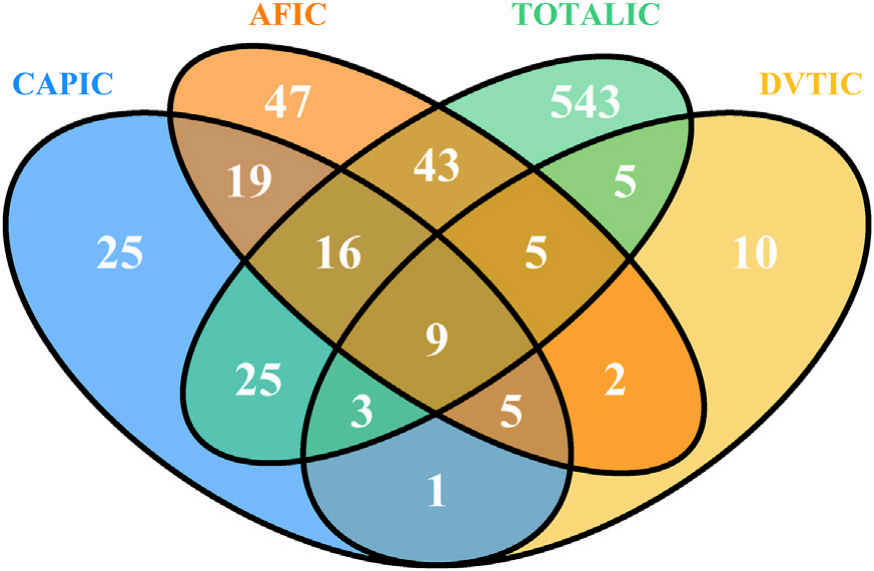
Venn diagram of the suspected AE signals associated with rivaroxaban detected using BCPNN algorithms at the PT level. Abbreviations: CAP: Cerebrovascular accident prophylaxis; AF: Atrial fibrillation; DVT: Deep vein thrombosis; TOTAL: All indications of rivaroxaban; IC, a value named information component in BCPNN algorithm.

**FIGURE 6:**
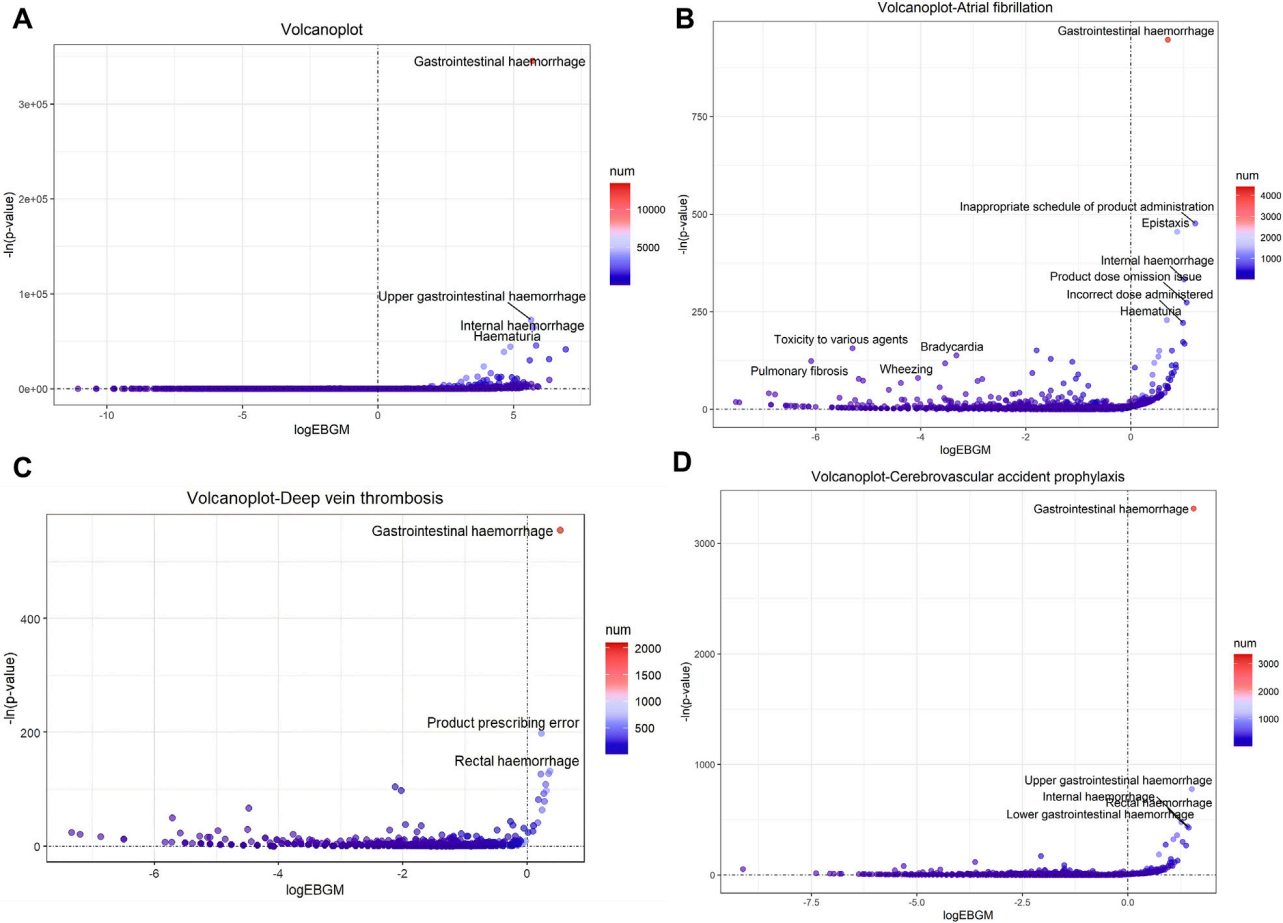
Signals of suspected AEs associated with rivaroxaban using MGPS algorithm at the PT level. **(A)** Volcano plot of detected AEs related to all indications **(B)** Volcano plot of detected AEs related to Atrial fibrillation **(C)** Volcano plot of detected AE related to Deep vein thrombosis **(D)** Volcano plot of detected AE related to Cerebrovascular accident prophylaxis. The *x*-axis denotes the logarithm of the reporting ROR (log_2_ (ROR)) or PRR (log_2_ (ROR)) or IC lower limit (log_2_(IC)) or EBGM lower limit (log_2_ (EBGM)). The *y*-axis is the negative logarithm of the *P* value calculated using the chi-square test (−ln (*P* value)). Positive values in the direction of the *y*-axis represent significant differences. The colors of the points represent the difference in the logarithm of the number of each ADR. In this scatterplot, the point in the upper right corner has a greater signal. The blue-to-red colors represent the number of times an adverse effect was reported. Abbreviations: EBGM, a value named empirical Bayesian geometric mean in MGPS algorithm; MGPS, multi-item gamma Poisson shrinker.

The top 10 signals sorted by the number of drug–adverse event combinations associated with three indications at the PT level in FAERS are exhibited in Table 5. Notably, regardless of indication, “Gastrointestinal haemorrhage”, “Epistaxis”, “Haematuria”, “Rectal haemorrhage”, and “Upper gastrointestinal haemorrhage” (Underline fonts) were detected as the most common and significant signals of suspected adverse events. Among Atrial fibrillation, Cerebrovascular accident prophylaxis and Deep vein thrombosis and the total reports of rivaroxaban, 9 signals were collectively identified using BCPNN algorithm, as shown in Figure 5. Volcano plots were used to visualise information intuitively on suspected adverse reactions by MGPS algorithm under the three indications and the total reports of rivaroxaban. The *x*-axis denotes the logarithm of the reporting EBGM lower limit, and the *y*-axis denotes the negative logarithm of the *P* value calculated using the chi-square test (-ln (*P* value)). Positive values in the direction of the *y*-axis represent significant differences. The colors of the points represent the difference in the logarithm of the number of each ADR. In this scatterplot, the point in the upper right corner indicates a stronger signal. The blue-to-red colors represent the number of times of reported adverse effects. Each label represents a specific PT. Based on the plots, “Gastrointestinal Hemorrhage” were identified jointly as the most significant signal (Figure 6).

**TABLE 5:** The top 10 signals sorted by the number of drug–adverse event combinations associated with three indications at the PT level.

Indication	PT	Drug-AE count	ROR	ROR 95%CI	PRR	PRR 95%CI	Chi-square	IC	IC 95%CI	IC signal strength	EBGM	EBGM 95%CI	IME important medical event)
Atrial fibrillation	Gastrointestinal haemorrhage	4,420	2.500	2.395–2.608	2.387	2.293–2.486	1886.061	0.767	0.714–0.820	+	1.702	1.631–1.776	Y
	Epistaxis	1,248	3.477	3.189–3.791	3.425	3.144–3.730	902.019	1.007	0.903–1.108	+	2.010	1.844–2.192	N
	Cerebrovascular accident	1,057	1.850	1.707–2.004	1.834	1.695–1.985	232.000	0.562	0.455–0.667	+	1.477	1.363–1.6	Y
	Haematuria	960	2.588	2.363–2.835	2.563	2.342–2.804	450.808	0.818	0.702–0.931	+	1.763	1.61–1.931	N
	Haemorrhage	959	1.324	1.223–1.433	1.319	1.22–1.426	48.683	0.272	0.162–0.379	+	1.207	1.115–1.307	N
	Rectal haemorrhage	951	2.107	1.931–2.299	2.089	1.917–2.277	294.228	0.667	0.552–0.778	+	1.587	1.455–1.732	Y
	Anaemia	910	0.907	0.84–0.979	0.909	0.843–0.980	6.204	−0.100	−0.208–0.008	-	0.933	0.864–1.008	N
	Upper gastrointestinal haemorrhage	891	2.068	1.89–2.261	2.051	1.878–2.241	263.647	0.652	0.534–0.767	+	1.572	1.437–1.719	Y
	Cerebral haemorrhage	842	1.802	1.648–1.970	1.790	1.639–1.956	171.262	0.542	0.422–0.659	+	1.456	1.332–1.592	Y
	Acute kidney injury	826	1.432	1.314–1.562	1.426	1.31–1.553	67.229	0.344	0.225–0.460	+	1.269	1.164–1.384	Y
Cerebrovascular accident prophylaxis	Gastrointestinal haemorrhage	3,342	9.781	9.159–10.446	8.724	8.193–9.29	6,617.709	1.656	1.588–1.721	++	3.151	2.95–3.365	Y
	Upper gastrointestinal haemorrhage	724	11.149	9.591–12.959	10.884	9.375–12.636	1,547.588	1.738	1.589–1.874	++	3.335	2.869–3.877	Y
	Epistaxis	716	2.496	2.265–2.751	2.458	2.234–2.704	362.947	0.882	0.75–1.008	+	1.843	1.672–2.031	N
	Rectal haemorrhage	660	5.739	5.063–6.506	5.626	4.971–6.369	948.308	1.45	1.301–1.588	+	2.732	2.41–3.097	Y
	Haematuria	604	4.128	3.664–4.65	4.06	3.609–4.567	637.302	1.255	1.104–1.397	+	2.387	2.119–2.689	N
	Acute kidney injury	576	4.81	4.235–5.463	4.731	4.171–5.366	710.455	1.351	1.194–1.497	+	2.551	2.246–2.898	Y
	Cerebrovascular accident	506	1.08	0.976–1.195	1.078	0.977–1.191	2.223	0.083	−0.061–0.226	-	1.059	0.958–1.172	Y
	Fall	494	0.861	0.779–0.952	0.864	0.783–0.953	8.63	−0.166	−0.309–0.023	-	0.892	0.807–0.985	N
	Cerebral haemorrhage	444	1.853	1.649–2.081	1.839	1.639–2.063	111.073	0.625	0.463–0.782	+	1.543	1.373–1.733	Y
	Internal haemorrhage	442	9.378	7.826–11.238	9.245	7.722–11.067	872.058	1.679	1.488–1.85	+	3.201	2.671–3.836	Y
Deep vein thrombosis	Gastrointestinal haemorrhage	2089	5.698	5.08–6.39	5.302	4.741–5.929	1,103.824	0.694	0.608–0.777	+	1.617	1.442–1.814	Y
	Deep vein thrombosis	1,046	1.531	1.383–1.694	1.508	1.368–1.663	68.863	0.249	0.135–0.361	+	1.188	1.074–1.315	Y
	Haematuria	601	3.102	2.618–3.674	3.051	2.58–3.606	189.969	0.548	0.389–0.702	+	1.462	1.235–1.732	N
	Pulmonary embolism	574	1.291	1.135–1.469	1.284	1.132–1.458	15.083	0.159	0.006–0.308	+	1.116	0.981–1.27	Y
	Rectal haemorrhage	567	4.579	3.736–5.612	4.497	3.675–5.503	258.064	0.657	0.49–0.817	+	1.577	1.286–1.933	Y
	Haemorrhage	512	1.179	1.032–1.347	1.175	1.031–1.339	5.869	0.105	−0.056–0.262	-	1.075	0.941–1.229	N
	Product prescribing error	499	21.52	13.901–33.314	21.107	13.645–32.65	390.335	0.858	0.672–1.033	+	1.812	1.171–2.805	N
	Upper gastrointestinal haemorrhage	445	6.474	4.96–8.449	6.376	4.891–8.311	249.344	0.729	0.537–0.911	+	1.657	1.27–2.163	Y
	Epistaxis	439	2.818	2.327–3.411	2.785	2.305–3.366	122.997	0.518	0.332–0.696	+	1.432	1.183–1.733	N
	Thrombosis	365	0.97	0.836–1.127	0.971	0.838–1.125	0.156	−0.02	−0.208–0.163	-	0.986	0.849–1.145	Y

## Discussion

Our study found that suspected adverse events related to rivaroxaban were most frequently reported among individuals aged 65 years and older, accounting for 55% of the adverse reaction reports, totaling 42,613 case. This was roughly similar to the age of the seven patients with adverse reaction reports associated with rivaroxaban covered in the preamble. It is worth mentioning that we found 60 commonly reported indications for rivaroxaban to be “Atrial fibrillation”, “Cerebrovascular accident prophylaxis”, “Deep vein thrombosis”, “Pulmonary embolism”and “Thrombosis prophylaxis”, which is similar to the therapeutic indications of rivaroxaban according to the European medicines agency’s production information. According to the baseline characteristics, we also found that people over 65 years old had the largest number of adverse reaction reports, which is consistent with epidemiological data that older adults are more likely to develop deep vein thrombosis and venous thromboembolism (Bulger et al., 2004; Wollenman et al., 2024).

Rivaroxaban is used for the prevention and treatment of deep vein thrombosis and pulmonary embolism formation in adults, as well as for the prevention of stroke and non- Central Nervous System embolism in patients with atrial fibrillation and in patients with non-valvular atrial fibrillation, and also for the treatment of hepatitis C. Based on this, the data mining results also showed that the suspected AE signals of “Surgical and medical surgery” and “Heart disease” were robust, because rivaroxaban is widely used in hip and knee replacement surgery and anticoagulation therapy for patients with nonvalvular atrial fibrillation. All these further indicate that our results are credible. And combined with the results of signal mining of overall rivaroxaban adverse drug reaction reports, the three most used indications for rivaroxaban were analysed individually, it was found that “Gastrointestinal haemorrhage”, and “Upper gastrointestinal haemorrhage” were the most common and signal significant suspected adverse reactions (Specific information is detailed in bold in Tables 4, 5).

The year 2015 saw the highest number of adverse reaction reports for rivaroxaban, with 14,751 cases accounting for 19% of the total. This surge in reports might be closely related to the drug’s approval for use in the United States since 2011. The number of AE reports decreased each year. We speculated that this might be due to the emergence of an increasing number of oral anticoagulants gradually replacing rivaroxaban. Using signal mining, we also found that rivaroxaban was significantly associated with an increased risk of gastrointestinal adverse events, especially gastrointestinal hemorrhage. This result was consistent with the findings of other studies. Fernandez et al. summarized the drug–drug interactions of rivaroxaban with other drugs in a systematic review including 31 studies and 28 case reports. They found that the drug-drug interactions of rivaroxaban with other drugs resulted in an increased risk of hemorrhage or thromboembolic events (Fernandez et al., 2021). In a prospective study, Njuguna et al. reported that the use of rivaroxaban was associated with a higher frequency of bleeding. This association was observed by evaluating patients who were newly initiated on rivaroxaban or switched from warfarin to rivaroxaban for treating venous thromboembolism at the National Referral Hospital in Western Kenya (Njuguna et al., 2023). A pharmacovigilance analysis by Sun et al. of suspected hemorrhagic events following antithrombotic drug use, based on the FAERS database, indicated that rivaroxaban was more strongly associated with hemorrhage than were other antithrombotic drug monotherapies. The combination of rivaroxaban with a drug such as clopidogrel was associated with an earlier onset of hemorrhagic events (Sun et al., 2022). These researches manifested that rivaroxaban use was a prominent risk factor for gastrointestinal bleeding.

Our study provided a comprehensive summary of the suspected AEs associated with rivaroxaban by five algorithms based on two statistical principles, and used the latest data and visualization methods innovatively to show the results. This study may provide a basis and supplement for the rational and safe use and regulation and policing of the drug, which can fill up a little study gap on adverse reaction reports of rivaroxaban alone. However, the use of databases in this study had certain shortcomings: First, the spontaneous adverse event reporting system suffered from reporting bias (Zhu et al., 2024) and shortcomings such as omissions absence, misreporting, the lack of denominator and missing data (Kong et al., 2023). Key patient information such as gender, age, and medication initiation time were missing in some proportions, which could impact the results. The information in these reports reflects only the observations of the reporters. Second, it was probably unable to estimate true risk or assess incidence. For one thing, the data lacked meaningful denominators to exclude prevalent cases. For another thing, these reports originate from actual patients who have several concomitant conditions and are being treated with different medicines, isolating from the complex patient reality could cause confusion and produce inaccurate information. Confounding factors such as potential drug-drug interactions; preexisting diseases; comorbidities; acute versus chronic duration of treatment; synergistic, antagonistic, additive effects with other pharmacologic agents; and genetic predisposition to therapy response^29 30^ could not be fully controlled. Third, most of the dosage information was missing, and hence we did not consider the effect of dose, which may be an essential factor in the AEs of rivaroxaban (Sun et al., 2022). Moreover, the analyzed reports were filtered by “primary suspect drug”, and did not include concomitant medicines, which could miss some crucial drug interaction signals and became one of the limitations of our study. Fourth, the FAERS is open and free, consumers and healthcare professionals are encouraged to report adverse events, 53.84% of the reporters were consumers. This high percentage of non-medical staff reporting probably undermines the credibility of the results. The documentation of one or more outcomes in a report does not necessarily mean that the suspect product mentioned in the report was the cause of those outcomes. Although we used various data mining algorithms for our study, the submission of a report to the FAERS database does not imply that the information contained in the report has been medically confirmed, nor does it imply that the reporter acknowledges that the drug caused or contributed to the event. Additionally, the disproportionality analysis neither quantified risk nor existed causality, but only provided an estimation of the signal strength from statistically significant only, so the results of the data mining methods should be used with caution when extrapolating conclusions and a causal relationship (Shu et al., 2022). More clinical data are still needed to support this as evidence.

In recent years, direct oral anticoagulants have been essential in treating and preventing thromboembolic incidents (Bozic et al., 2023). Two clinical trials of rivaroxaban for the treatment and post-surgical prophylaxis of thrombosis in pediatric populations reported that rivaroxaban was safe and effective in the treatment and secondary prevention of venous thromboembolism in children (McCrindle et al., 2021; Bosch and Albisetti, 2022). However, a study including 236 cases of direct oral anticoagulant use in pregnancy showed that rivaroxaban was the most reported direct oral anticoagulant in pregnancy and that rivaroxaban resulted in a higher rate of miscarriage and a 4% rate of malformations (Lameijer et al., 2018). Future research should expand the exploration of patient populations using rivaroxaban, focusing not only on the elderly population but also on the child–adolescent population and women during pregnancy. In addition, if rivaroxaban is to be used safely and appropriately, clinicians and pharmacists must weigh the pros and cons, considering the patient’s age, sex, medical condition, physical condition, drug dose, combination of drugs, and many other factors (Mardi et al., 2023; Moldenhauer et al., 2023). Moreover, researchers also can be encouraged to better design their studies on databases, so that providing more information on studying concomitant drugs with this drug would be useful for the readers and for future researches. Some studies have shown that genes were also associated with the adverse effects of rivaroxaban, providing a reference and basis for individualized treatment (Kim et al., 2023; Li et al., 2023). With a large number of pharmacovigilance (PV) studies are currently being published vigorously, it is necessary that large PV database analyses must be conducted taking into consideration some basic aspects to start giving signals.

In conclusion, our study employs a combined application of ROR, PRR, MHRA, BCPNN, and MGPS algorithms with the aim of leveraging the strengths of each to broaden the detection scope, verify results from multiple perspectives, and make rational use of the distinctive features of different algorithms to detect more comprehensive and reliable suspected signals. “Gastrointestinal haemorrhage”, “Upper gastrointestinal haemorrhage” should be the focus of attention to prevent adverse events. And it is worth noting that uncommon but significantly ADE signals, such as “Coagulation factor X level increased”, “Basal ganglia haematoma”, and “Proctitis haemorrhagic” also should be concentrated on.

## Conclusions

This study provided an objective reference for pharmacovigilance by mining the safety signals of rivaroxaban. “Gastrointestinal disorders”, “Injury, poisoning, and procedural complications”, “Nervous system disorders” and “Vascular disorders” were the significant system organ classes with adverse events. Focus also should be placed on suspected AEs with strong signals not common reported, such as “Coagulation factor X level increased”, “Basal ganglia haematoma”, and “Proctitis haemorrhagic”. In addition, both the risk and benefit should be appropriately weighed when prescribing rivaroxaban for patients. Close attention should be paid to disease progression, and timely intervention measures should be taken against AEs to reduce the risk of poor outcomes.

## Data Availability

Publicly available datasets were analyzed in this study. These data can be found at: https://www.fda.gov/drugs/drug-approvals-and-databases/fda-adverse-event-reporting-system-faers. Further inquiries can be directed to the corresponding author.
