# Supplementary material for: Suspected adverse drug reactions of rivaroxaban reported in the United States food and drug administration adverse event reporting system database: a pharmacovigilance study
Source: Front Pharmacol. 2024 Sep 6;15:1399172. doi: 10.3389/fphar.2024.1399172 (PMC11412890; doi:10.3389/fphar.2024.1399172)
Supplement: Supplementary file 1 [file DataSheet1.docx]

**Supplementary information**

**Table S1.** Formulas for different algorithms.

| Algorithm | Calculation formulas | Signal criterions |
| --- | --- | --- |
| ROR | $ROR=\frac{(a/c)}{(b/d)}=\frac{ad}{bc}$  $SEln(ROR)=\sqrt{(\frac{1}{a}+\frac{1}{b}+\frac{1}{c}+\frac{1}{d})}$  $95\%CI=e^{ln(ROR)\pm1.96\sqrt{(\frac{1}{a}+\frac{1}{b}+\frac{1}{c}+\frac{1}{d})}}$ | a≥3 and 95% CI(lower limit) ＞1,  1< ROR -1.96SE<50 weak signal (+); 50≤ROR -1.96SE<1 000 medium intensity signal  (++); 1 000≤ROR -1.96SE high intensity signal (+++) |
| PRR | $PRR＝\frac{a/(a+b)}{c/(c+d)}$  $SE(lnPRR)=\sqrt{\frac{1}{a}-\frac{1}{a+b}+\frac{1}{c}-\frac{1}{c+d}}$  ${95\%CI==e}^{ln(PRR)\pm1.96\sqrt{\frac{1}{a}-\frac{1}{a+b}+\frac{1}{c}-\frac{1}{c+d}}}$ | a≥3 and 95% CI(lower limit) ＞ 1,  1<PRR-1.96SE<50 weak signal (+); 50≤PRR-1.96SE<1 000 medium intensity signal  (++); 1 000≤PRR-1.96SE high intensity signal (+++) |
| MHRA | $PRR＝\frac{a/(a+b)}{c/(c+d)}$  $\chi2 =\frac{{(ad-bc)}^{2}(a+b+c+d)}{( a+b)(a+c)(c+d)(b+d)}$ | a≥3 and PRR≥2 and 4, 1<PRR-1.96SE<50 weak signal (+); 50≤PRR-1.96SE<1 000 medium intensity signal  (++); 1 000≤PRR-1.96SE high intensity signal (+++) |
| BCPNN | ${IC=log}_{2}\frac{p(x,y)}{p(x)p(y)}={log}_{2}\frac{a(a+b+c+d)}{(a+b)(a+c)}$  ${E\left( IC \right)=log}_{2}\frac{(a+\gamma11)(a+b+c+d+\alpha)(a+b+c+d+\beta)}{（a+b+c+d+\gamma）(a+b+\alpha1)(a+c+\beta1)}$  $V(IC)=\frac{1}{{(ln2)}^{2}}\{\left[ \frac{\left( a+b+c+d \right)-a+\gamma-\gamma11}{\left( a+\gamma11 \right)\left( 1+a+b+c+d+\gamma\right)} \right]+\left[ \frac{\left( a+b+c+d \right)-\left( a+b \right)+\alpha-\alpha1}{\left( a+b+\alpha1 \right)\left( 1+a+b+c+d+\alpha\right)} \right]+\left[ \frac{\left( a+b+c+d \right)-\left( a+c \right)+\beta-\beta1}{\left( a+c+\beta1 \right)\left( 1+a+b+c+d+\beta\right)} \right]\}$  $\gamma=\gamma11\frac{(a+b+c+d+\alpha)(a+b+c+d+\beta)}{(a+b+\alpha1)(a+c+\beta1)}$  $IC-2SD=E(IC)-2\sqrt{V(IC)}$  $Especially,\alpha1=\beta1=1；\alpha=\beta=2；\gamma11=1$ | According to the signal strength level:   1. no signal (-): IC-2SD ≤0； 2. weak signal （+）:0＜ IC-2SD ≤1.5； 3. middle signa（++）:1.5＜ IC-2SD ≤3； 4. strong signal（+++）: IC-2SD ＞3   Lower limit of confidence interval （IC-2SD）>0 |
| MGPS | $\mathrm{EBGM}＝\frac{a(a+b+c+d)}{(a+c)(a+b)}$  $95\%CI=e^{ln(EBGM)\pm1.96\sqrt{(\frac{1}{a}+\frac{1}{b}+\frac{1}{c}+\frac{1}{d})}}$ | EBGM05>2 |

**Table S2**. The proportion of adverse event reports under system organ class (SOC) with rivaroxaban in FAERS.

| **System organ class（SOC）** | **Number of Cases*** | **Proportion of** **Adverse Event Reports (%)**** |
| --- | --- | --- |
| Gastrointestinal disorders | 37084 | 20.90 |
| Injury, poisoning and procedural complications | 21524 | 12.13 |
| Nervous system disorders | 21205 | 11.95 |
| Vascular disorders | 14322 | 8.07 |
| General disorders and administration site conditions | 12425 | 7.00 |
| Respiratory, thoracic and mediastinal disorders | 11927 | 6.72 |
| Renal and urinary disorders | 8583 | 4.84 |
| Investigations | 7598 | 4.28 |
| Blood and lymphatic system disorders | 5937 | 3.35 |
| Skin and subcutaneous tissue disorders | 5777 | 3.26 |
| Musculoskeletal and connective tissue disorders | 5473 | 3.08 |
| Cardiac disorders | 5388 | 3.04 |
| Infections and infestations | 3553 | 2.00 |
| Reproductive system and breast disorders | 3403 | 1.92 |
| Psychiatric disorders | 2295 | 1.29 |
| Surgical and medical procedures | 1996 | 1.12 |
| Eye disorders | 1895 | 1.07 |
| Neoplasms benign, malignant and unspecified (incl cysts and polyps) | 1555 | 0.88 |
| Metabolism and nutrition disorders | 1503 | 0.85 |
| Hepatobiliary disorders | 1049 | 0.59 |
| Product issues | 902 | 0.51 |
| Congenital, familial and genetic disorders | 701 | 0.40 |
| Ear and labyrinth disorders | 477 | 0.27 |
| Immune system disorders | 325 | 0.18 |
| Social circumstances | 272 | 0.15 |
| Endocrine disorders | 213 | 0.12 |
| Pregnancy, puerperium and perinatal conditions | 83 | 0.05 |

*: Number of Cases means cases of drug-adverse event combination under organ system classification (SOC)；

**: Proportion of adverse event reports under organ system classification (SOC) = the number of adverse event reports under organ system classification (SOC)/total number of adverse event reports of target drugs.

**
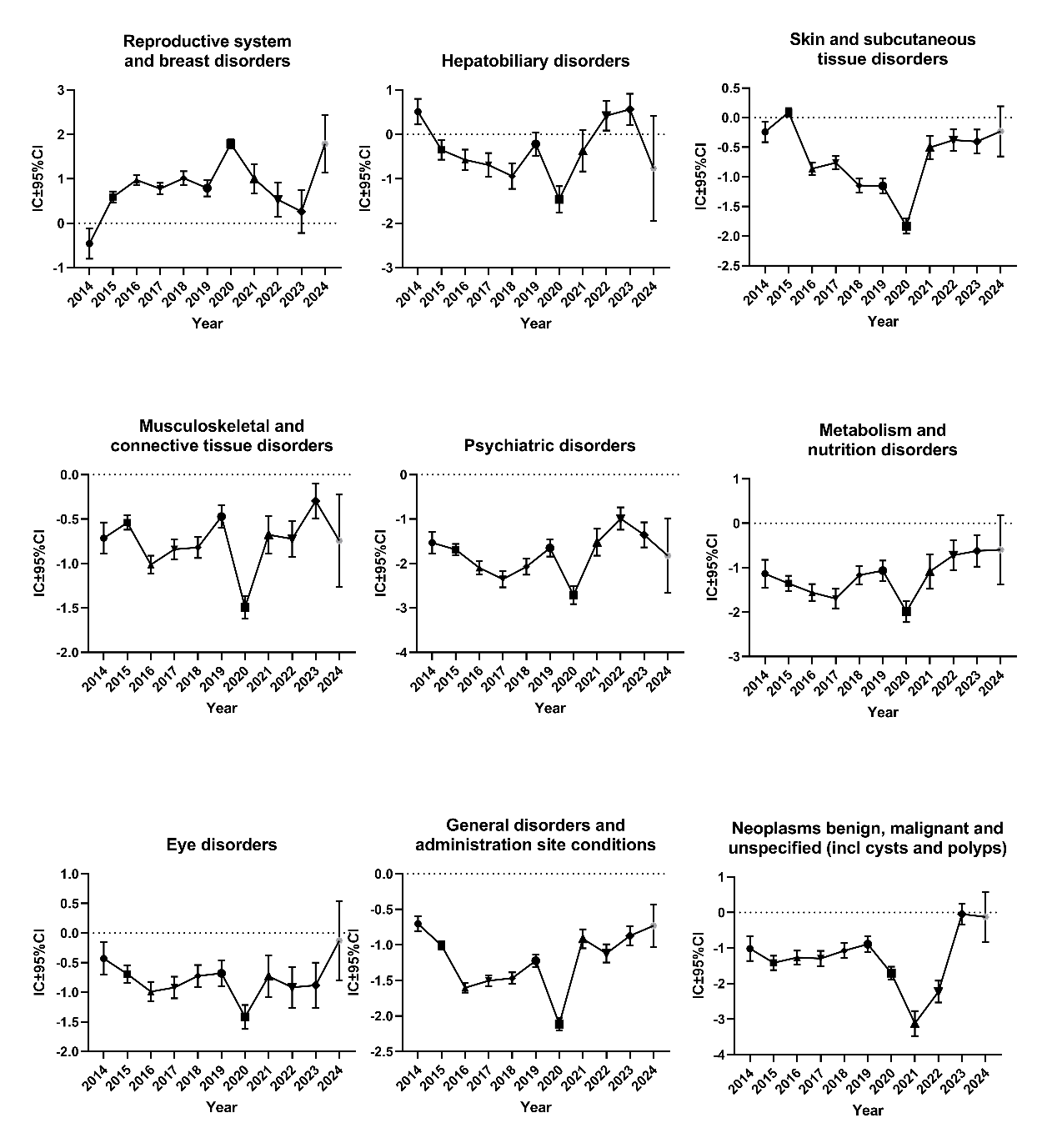
**

**Figure S1**. Information component and its 95% credibility interval over time for different system organ classes of rivaroxaban-associated adverse events.

Abbreviations: IC, information component; CI, credibility interval.

**
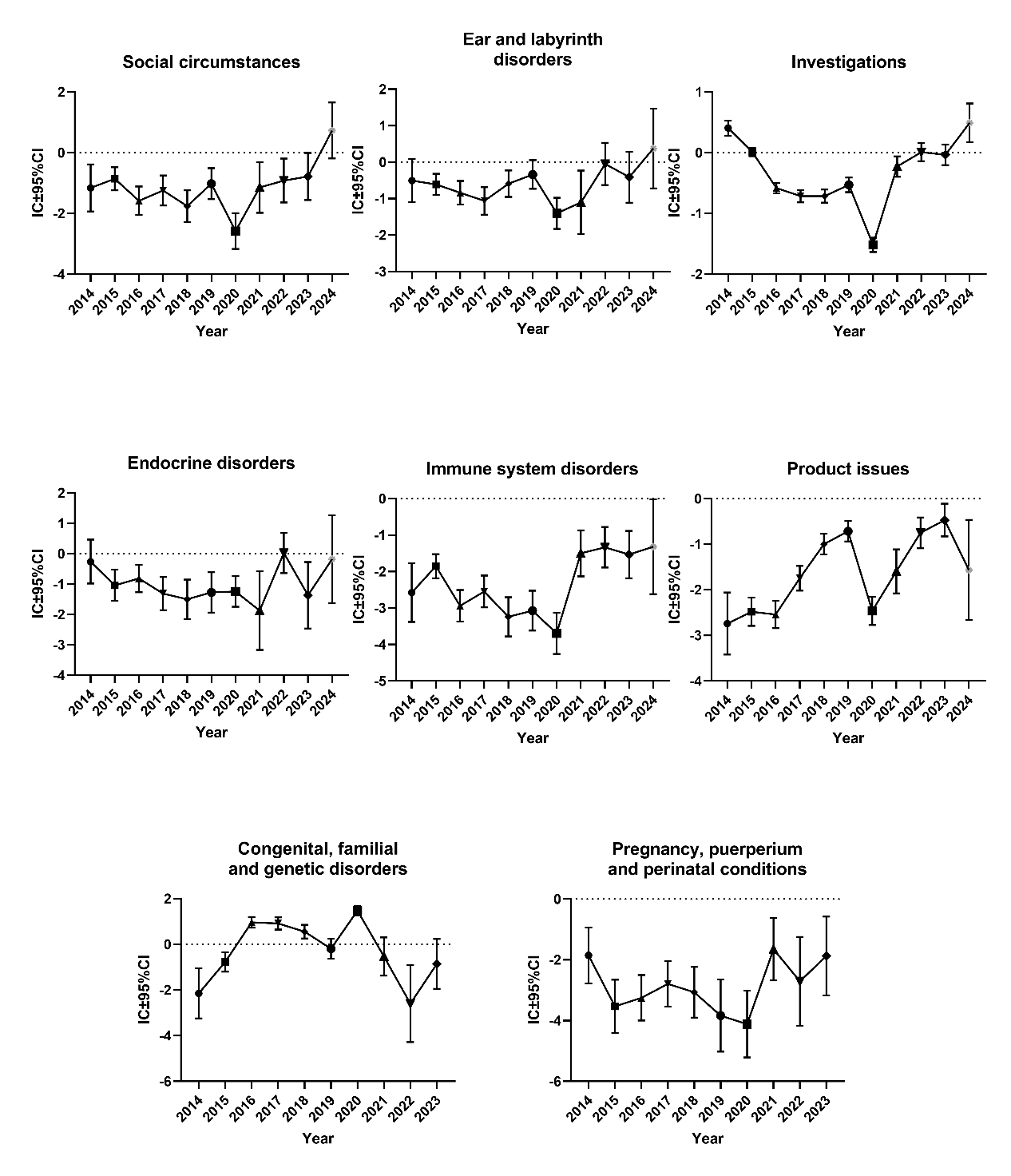
**

**Figure S2**. Information component and its 95% credibility interval over time for different system organ classes of rivaroxaban-associated adverse events.

Abbreviations: IC, information component; CI, credibility interval.


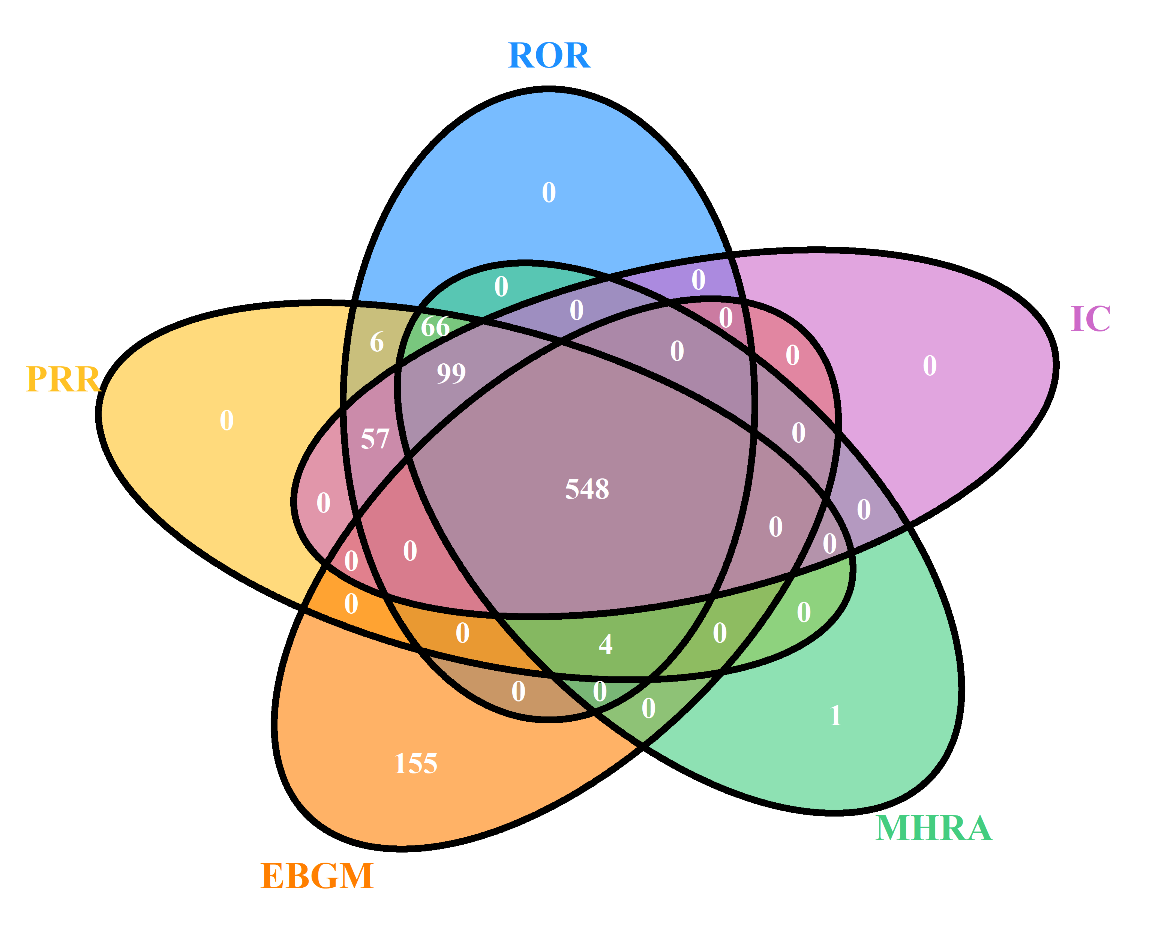


**Figure S3**. The Venn diagram for signals of AE reports of rivaroxaban by ROR, MHRA, EBGM and BCPNN algorithms.

Abbreviations: ROR, reporting odds ratio; MHRA, a≥3 and PRR≥2 and χ2≥4; IC, a value named information component in BCPNN algorithm; BCPNN, Bayesian confidence propagation neural network; EBGM, a value named empirical Bayesian geometric mean in MGPS algorithm; MGPS, multi-item gamma Poisson shrinker.

**
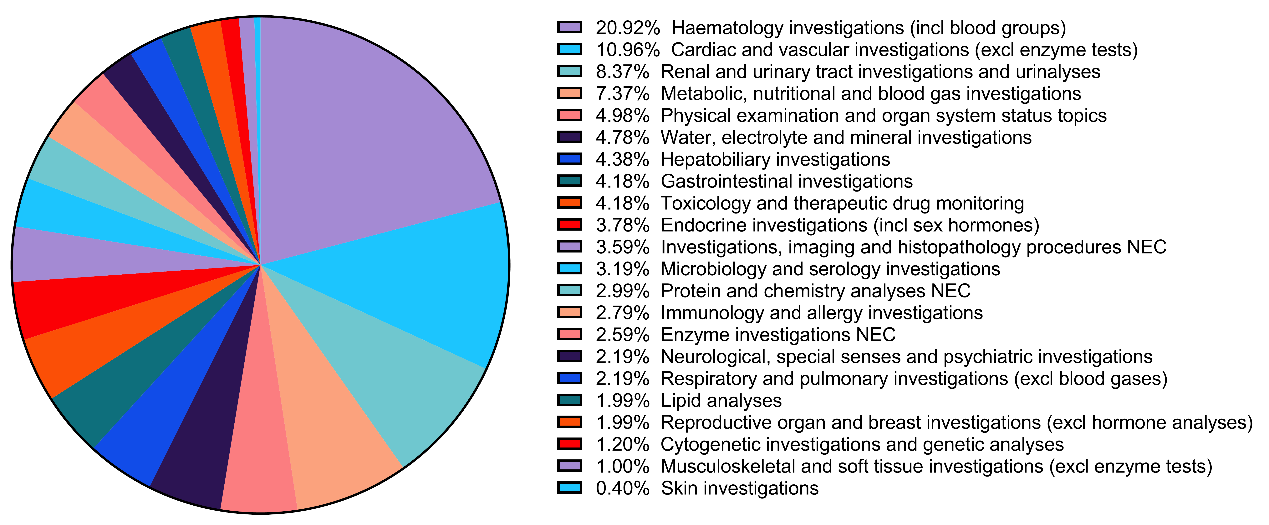
**

**Figure S4**. The pie chart of every HLGT under the “Investigations” category.

HLGT: High Level Group Term.

**
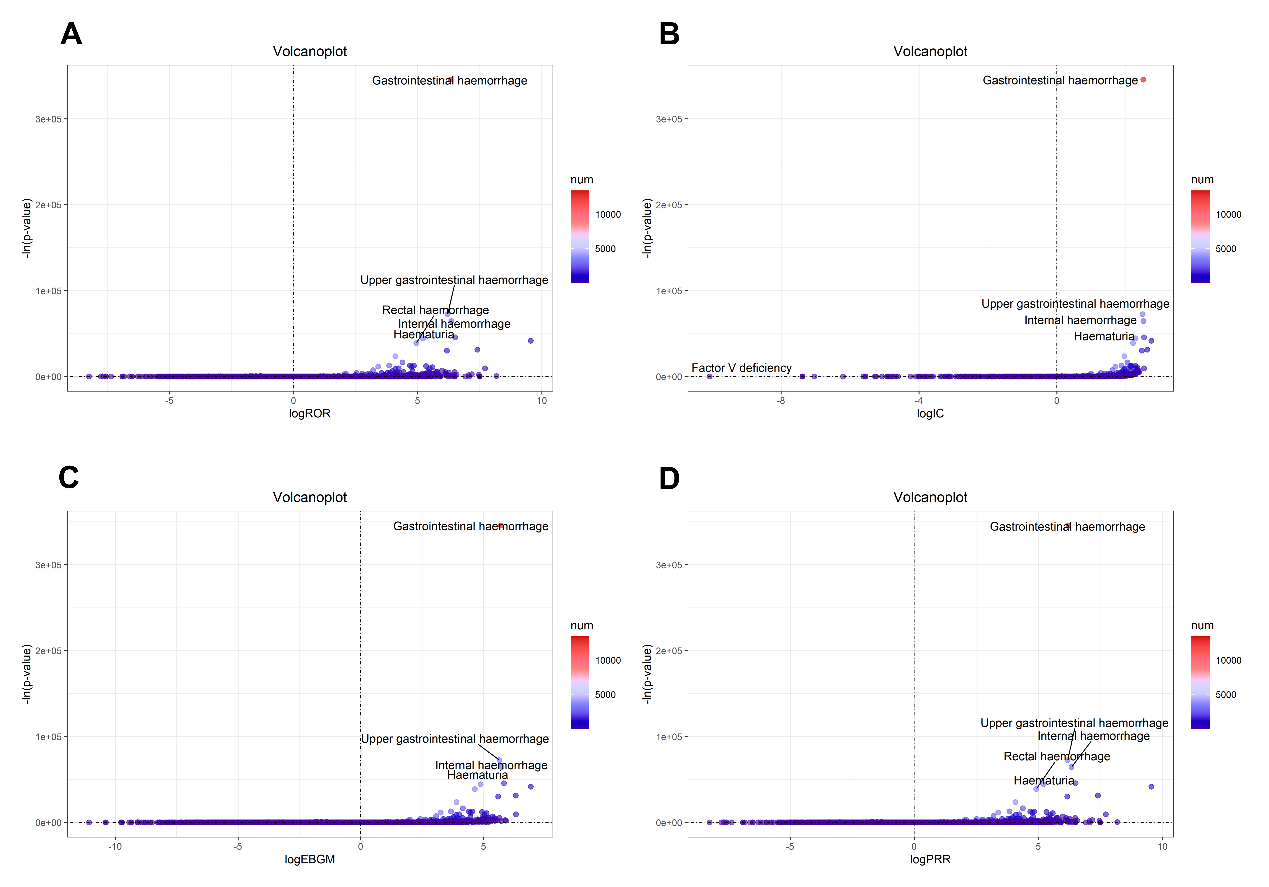
**

**Figure S5. Rivaroxaban associated with signals of AE reports.**

(A) Volcano plot of AE detected using ROR algorithm at the PT level. (B) Volcano plot of AE detected using BCPNN algorithm at the PT level; (C) Volcano plot of AE detected using MGPS algorithm at the PT level. (D) Volcano plot of AE detected using PRR algorithm at the PT level. The x-axis denotes the logarithm of the reporting ROR (log2(ROR)) or PRR (log2(ROR)) or IC lower limit (log2(IC)) or EBGM lower limit (log2(EBGM)). The y-axis is the negative logarithm of the P value calculated using the chi-square test (−ln(P value)). Positive values in the direction of the y-axis represent significant differences. The colors of the points represent the difference in the logarithm of the number of each ADR. In this scatterplot, the point in the upper right corner has a greater signal. The blue-to-red colors represent the number of times an adverse effect was reported.

Abbreviations: ROR, reporting odds ratio; IC, a value named information component in BCPNN algorithm; BCPNN, Bayesian confidence propagation neural network; EBGM, a value named empirical Bayesian geometric mean in MGPS algorithm; MGPS, multi-item gamma Poisson shrinker; PRR, proportional reporting ratio.
